# Supplementary material for: The causal role of circulating inflammatory markers in osteoporosis: a bidirectional Mendelian randomized study
Source: Front Immunol. 2024 Jul 18;15:1412298. doi: 10.3389/fimmu.2024.1412298 (PMC11291241; doi:10.3389/fimmu.2024.1412298)
Supplement: Supplementary file 1 [file DataSheet_1.zip › supplementary material/OSTEOPOROSIS-CIM/GCST90274838.tsv.gz_leave_one_out.pdf]

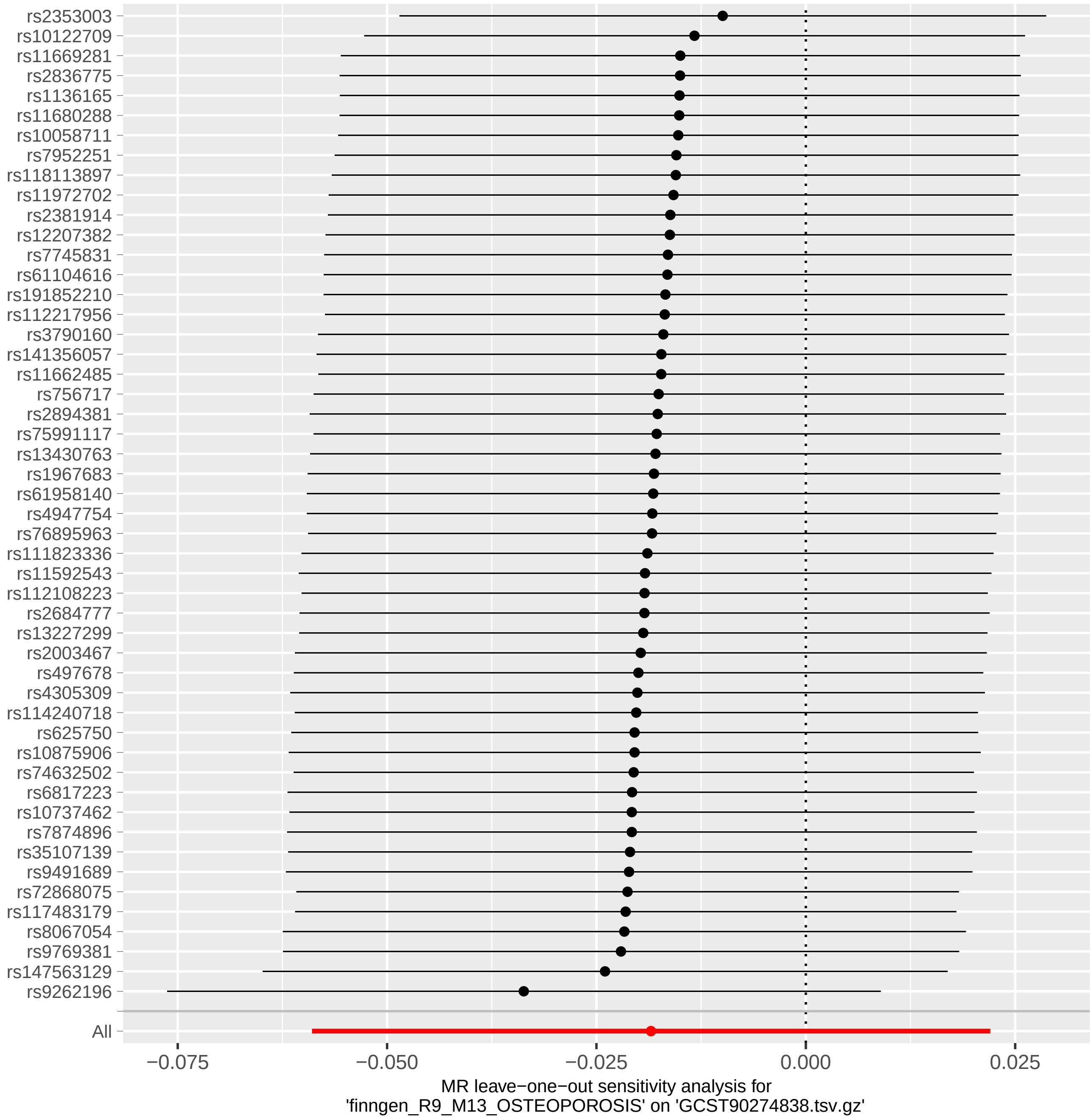
rs2353003
rs10122709
rs11669281
rs2836775
rs1136165
rs11680288
rs10058711
rs7952251
rs118113897
rs11972702
rs2381914
rs12207382
rs7745831
rs61104616
rs191852210
rs112217956
rs3790160
rs141356057
rs11662485
rs756717
rs2894381
rs75991117
rs13430763
rs1967683
rs61958140
rs4947754
rs76895963
rs111823336
rs11592543
rs112108223
rs2684777
rs13227299
rs2003467
rs497678
rs4305309
rs114240718
rs625750
rs10875906
rs74632502
rs6817223
rs10737462
rs7874896
rs35107139
rs9491689
rs72868075
rs117483179
rs8067054
rs9769381
rs147563129
rs9262196
All
−0.075
−0.050
−0.025
0.000
0.025
MR leave−one−out sensitivity analysis for
'finngen_R9_M13_OSTEOPOROSIS' on 'GCST90274838.tsv.gz'
